# Efficacy of combination therapy with minocycline for treating *Stenotrophomonas maltophilia* infections

**DOI:** 10.1093/jacamr/dlag224

**Published:** 2026-10-16

**Authors:** Tuvanont Pongdumbun, Yong Rongrungruang, Adhiratha Boonyasiri

**Affiliations:** Department of Internal Medicine, Golden Jubilee Medical Center, Faculty of Medicine, Siriraj Hospital, Mahidol University, Bangkok, Thailand; Division of Infectious Diseases and Tropical Medicine, Department of Medicine, Faculty of Medicine, Siriraj Hospital, Mahidol University, Bangkok, Thailand; Clinical Epidemiology Unit, Department of Research and Development, Faculty of Medicine, Siriraj Hospital, Mahidol University, Bangkok, Thailand

## Abstract

**Background:**

*Stenotrophomonas maltophilia* is a multidrug-resistant opportunistic pathogen associated with substantial in-hospital mortality. However, data supporting the efficacy of treating *S. maltophilia* infections using a combination of antimicrobials remain inconclusive.

**Objectives:**

To assess whether adjunctive minocycline improves clinical outcomes of *S. maltophilia* infections compared with standard monotherapy.

**Methods:**

This randomized, double-blind, placebo-controlled trial was conducted at Siriraj Hospital, Thailand. Adults with confirmed *S. maltophilia* infections were randomized to receive minocycline or a matching placebo in addition to monotherapy with either levofloxacin or trimethoprim/sulfamethoxazole.

**Results:**

This trial enrolled 112 patients (71.4% [*n* = 80/112] of patients requiring an intensive care unit [ICU] and 66.1% [*n* = 74/112] of patients were diagnosed with ventilator-associated pneumonia [VAP]). Baseline demographic and clinical characteristics were balanced between intervention and control groups. Adjunctive minocycline did not significantly reduce 28-day-mortality (*n* = 24/55[43.6%] vs *n* = 22/57[38.6%], *P* = 0.590) but did achieve significantly higher microbiological response rates at early assessment (*n* = 25/55[45.5%] vs *n* = 8/57[14.0%], *P* < 0.001) and the end of treatment (*n* = 44/55[80.0%] vs *n* = 32/57[56.1%], *P* = 0.023). In contrast, clinical response rates did not differ between groups at either 72 h after the start of study treatment or the end of study treatment. In multivariable analysis, two factors were independently associated with 28-day mortality: history of antibiotic use before admission (adjusted odds ratio [aOR]: 7.65, 95% confidence interval [CI]: 2.04–28.76; *P* = 0.030) and septic shock (aOR: 7.24, 95% CI: 2.75–19.23; *P* < 0.001).

**Conclusions:**

Although adding minocycline to either levofloxacin or trimethoprim/sulfamethoxazole therapy enhanced microbiological eradication, it did not confer a measurable survival or clinical advantage. Combination therapy was well tolerated. This study is potentially of interest to those treating ICU patients, as the majority of infections were VAP.

## Introduction

*Stenotrophomonas maltophilia* is a Gram-negative, multidrug-resistant pathogen and an important cause of hospital-acquired infections, particularly bloodstream and pulmonary infections in patients with multiple comorbidities.^1,2^  *Stenotrophomonas maltophilia* infections are associated with high mortality rates, ranging from 21% to 69%.^3^ Risk factors for *S. maltophilia* infection include broad-spectrum antibiotic use, prolonged hospitalization, intensive care unit (ICU) stay, mechanical ventilation, central venous catheterization and immunocompromised status.^4,5^ Due to the complexity of resistance mechanisms,^6,7^ treatment guidelines from the Infectious Diseases Society of America (IDSA)^8^ and the European Society of Clinical Microbiology and Infectious Diseases^9^ recommend treating severe infections with a combination of antibiotics, with trimethoprim/sulfamethoxazole (TMP/SMX) as the mainstay of treatment. The 2024 IDSA guidance states that minocycline is a preferred component of combination therapy due to its favourable *in vitro* characteristics, tolerability, and the availability of susceptibility breakpoints. One study conducted at Siriraj Hospital (Bangkok, Thailand) between 2013 and 2016 found that the susceptibility rates of *S. maltophilia* to minocycline, levofloxacin and TMP/SMX were 100%, 94% and 91%, respectively.^10^ However, that study showed a mortality rate of 54% with TMP/SMX as the first-line treatment, followed by levofloxacin as the second-line treatment. Although TMP/SMX is the recommended first-line treatment, a high incidence of dose-related adverse events and an increasing trend of resistance in recent clinical practice have prompted the more frequent use of levofloxacin for treating *S. maltophilia* infections.^11^

Minocycline, a tetracycline-class antibiotic used to treat respiratory and urinary tract infections, is effective against *S. maltophilia* infections.^12^ In Thailand, minocycline is only available in oral form as 50 mg capsules. Minocycline exhibits good oral absorption, with bioavailability of 95%–100%^13^ and no reported food–drug interactions. A systematic review evaluating the use of minocycline for lower respiratory tract infections found that it was effective in over 80% of cases (four studies involving 28 patients).^14^ The minocycline dose administered was uniformly 100 mg either orally or intravenously twice daily, and no serious adverse effects were reported. According to the Clinical and Laboratory Standards Institute (CLSI) M100 guidelines (34th edition), the minimum inhibitory concentration breakpoint for *S. maltophilia* susceptibility to minocycline is ≤1 mg/L for the susceptible strain. Studies have shown that *S. maltophilia* is more susceptible to minocycline (99.5%) than to TMP/SMX (93.0%).^15^

Despite its efficacy, to our knowledge, only observational studies have addressed the combined use of minocycline with other antibiotics for treating *S. maltophilia* infections. The mortality rate from *S. maltophilia* infections at Siriraj Hospital (54%)^10^ is much higher than that reported in other countries (24.0%–36.6%).^4,16^ Therefore, further randomized controlled studies are needed to evaluate the effectiveness of minocycline combination therapy. Consequently, this randomized, double-blind, placebo-controlled trial aimed to compare 28-day all-cause mortality, clinical and microbiological responses, and adverse events of standard monotherapy versus combination therapy with minocycline for *S. maltophilia* infections.

## Materials and methods

### Study design and population

This was a randomized, double-blind, placebo-controlled, parallel-group superiority trial designed to evaluate whether adjunctive oral minocycline, when added to standard monotherapy, was superior to standard monotherapy alone in reducing 28-day all-cause mortality among hospitalized patients with *S. maltophilia* infection. This trial was conducted at Siriraj Hospital, a 2300-bed tertiary care university hospital in Bangkok, Thailand, between November 2022 and January 2024. The trial protocol was approved by the Institutional Review Board at Siriraj Hospital, and all patients or their legal representatives provided written informed consent to participate in this trial. All aspects of this trial complied with the principles and guidelines outlined in the 1964 Declaration of Helsinki and all its subsequent amendments. The study was registered under clinicaltrials.gov (NCT05575427).

The inclusion criteria were (i) aged 18–95 years, (ii) hospitalized with a laboratory-confirmed *S. maltophilia* infection, (iii) able to take or tolerate enteral nutrition (oral or tube feeding) and (iv) indicated for potential intravenous or enteral treatment with TMP/SMX or levofloxacin within 96 h before enrolment. Because respiratory isolation of *S. maltophilia* may reflect either colonization or true infection, the diagnosis of *S. maltophilia* pneumonia was determined by the primary investigator and attending physician based on the overall clinical context, rather than microbiological culture results alone. Patients were considered eligible when the isolate was clinically judged to represent an infection requiring antimicrobial treatment.

The exclusion criteria were (i) pregnancy or breastfeeding, (ii) major contraindication to minocycline or both TMP/SMX and levofloxacin (drug allergy), (iii) *S. maltophilia* infection in the urinary tract, (iv) liver impairment (≥3 times the upper limit of aspartate aminotransferase (AST) or alanine aminotransferase (ALT), (v) current use of antibiotics in the same class as minocycline (tigecycline and doxycycline) and (vi) moribundity (life expectancy of <24 h, as assessed by the primary physician or investigators). Patients with septic shock were not excluded if the attending physician and investigators assessed them as being able to tolerate enteral nutrition. For all participants, *S. maltophilia* isolates were identified, and their antimicrobial susceptibility profiles to minocycline, levofloxacin and TMP/SMX were determined according to CLSI recommendations.

### Randomization and study treatments

Participants were randomized in blocks of four to receive monotherapy with either TMP/SMX or levofloxacin or combination therapy with oral minocycline in addition to TMP/SMX or levofloxacin. Randomization was performed using a computer-generated list of random assignments from a web-based randomization tool (Sealed Envelope Ltd., London, UK). The investigators and attending physician were unaware of participants’ group allocation after randomization. The primary investigator performed all analyses, and group allocations were not disclosed until the last enrolled patient had completed the 28-day follow-up.

Participants randomized to combination therapy received oral minocycline at a loading dose of 200 mg (four capsules) followed by a maintenance dose of 100 mg (two capsules), along with TMP/SMX (80 mg TMP + 400 mg SMX tablet) or levofloxacin (500 or 750 mg tablet). Participants randomized to monotherapy received TMP/SMX (80 mg TMP + 400 mg SMX tablet) or levofloxacin (500 or 750 mg tablet). The total treatment duration for combination therapy ranged from 7 to 28 days depending on the site, infection severity and overall patient response. Dosage was adjusted based on renal function as needed (Tables S1 and S2, available as Supplementary data at *JAC-AMR* Online). Both medications were discontinued simultaneously at the end of study treatment.

### Outcomes and definitions

A flow diagram of the trial protocol is presented in Figure S1. Participants underwent daily clinical assessments from enrolment until the end of study treatment, death or hospital discharge, whichever occurred first. The primary efficacy endpoint was 28-day all-cause mortality. Secondary endpoints included clinical response at 72 h and at the end of treatment, as well as microbiological response at 72 h and at the end of treatment.

Clinical outcomes were assessed at day 3 after initiation of the study treatment, at the end of the study treatment, and 28 days after treatment with the study drug(s). Clinical outcomes were classified as favourable (complete resolution or significant improvement of the signs and symptoms present at baseline and no new symptoms, such that no additional antimicrobial therapy is warranted) or unfavourable (no complete resolution or significant improvement of the signs and symptoms present at baseline, or new symptoms have developed and require the initiation of a non-study antimicrobial therapy, as well as death or intolerance to study drug[s] leading to discontinuation of the study treatment).

Microbiological culture specimens were obtained from the infection site on Day 3 after initiating the study treatment and then again at the end of study treatment, this in addition to the baseline sample collected to determine the microbiological outcomes. The microbiological outcomes were classified as eradication (*S. maltophilia* is not detected, and no new pathogen is detected), persistence (the same *S. maltophilia* strain from the previous culture is detected, with no new pathogen detected) and new pathogen (a pathogen species different from the previous culture is detected).

Liver and renal function tests were performed at least twice per week. Acute kidney injury (AKI) was determined as at least the injury category of the RIFLE (risk, injury, failure, loss of kidney function and end-stage kidney disease) classification.^17^ Acute hepatitis was defined as an increase of ≥5 times the upper limit of normal for AST and/or ALT, or symptomatic patients with an increase of ≥3 times the upper limit of normal for AST and/or ALT.

### Sample size estimation and statistical analysis

In a retrospective study conducted at Siriraj Hospital, the in-hospital mortality rate of patients with *S. maltophilia* infections treated with antibiotic monotherapy was 54%.^10^ In comparison, in a study conducted in the USA, 30-day infection-related mortality of patients with *S. maltophilia* infections treated with combination therapy was 15.8%.^18^ Assuming that combination therapy would decrease the mortality rate by 50% (from 54% to 27%), and allowing for 5% type I error and 20% type II error rates, the minimum sample size required was estimated to be 51 per group. To account for possible dropouts, the sample size was increased by 20% in each group. Therefore, the target was 62 patients per group, with a total sample size of 124. This assumed effect size was informed by mortality estimates reported in previous observational studies; however, these studies were conducted in different healthcare settings and used different mortality definitions and assessment time points.

All statistical analyses were performed using the SPSS Statistics for Windows (version 20; IBM Corp., Armonk, NY, USA), and *P* values <0.05 were considered statistically significant. Categorical variables are presented as numbers (percentages) and were compared between groups using the chi-squared test or Fisher’s exact test. Continuous variables are presented as means ± standard deviations (SDs; if normally distributed) or medians (interquartile ranges [IQRs]; if normally distributed) and were compared between groups using Student’s t-test (if normally distributed) or the Mann–Whitney *U* test (if non-normally distributed). For the primary outcome, we estimated the risk ratio (RR) and absolute risk difference (RD), with corresponding two-sided 95% confidence intervals (CIs), comparing the combination therapy group with the monotherapy group. Risk factors significantly and independently associated with the primary endpoint (28-day mortality) were identified using univariate analysis and multivariate logistic regression. The univariate analysis results are reported as crude odds ratios (ORs) with 95% CIs, and the multivariate regression results are reported as adjusted ORs (aORs) with 95% CIs. All randomized participants were analysed according to their assigned treatment group, regardless of treatment adherence or subsequent treatment modifications, following the intention-to-treat principle. No data were missing for the primary outcome or for any secondary outcomes; therefore, no imputation was performed.

## Results

### Participants’ baseline characteristics

This trial enrolled 112 patients with *S. maltophilia* infections, of whom 55 were randomized to the combination therapy group and 57 to the monotherapy group (Figure 1). The primary reason for exclusion of otherwise eligible patients was the treating physician’s decision not to treat the *S. maltophilia* infection (1437/2447, 58.7%).

**Figure 1.**
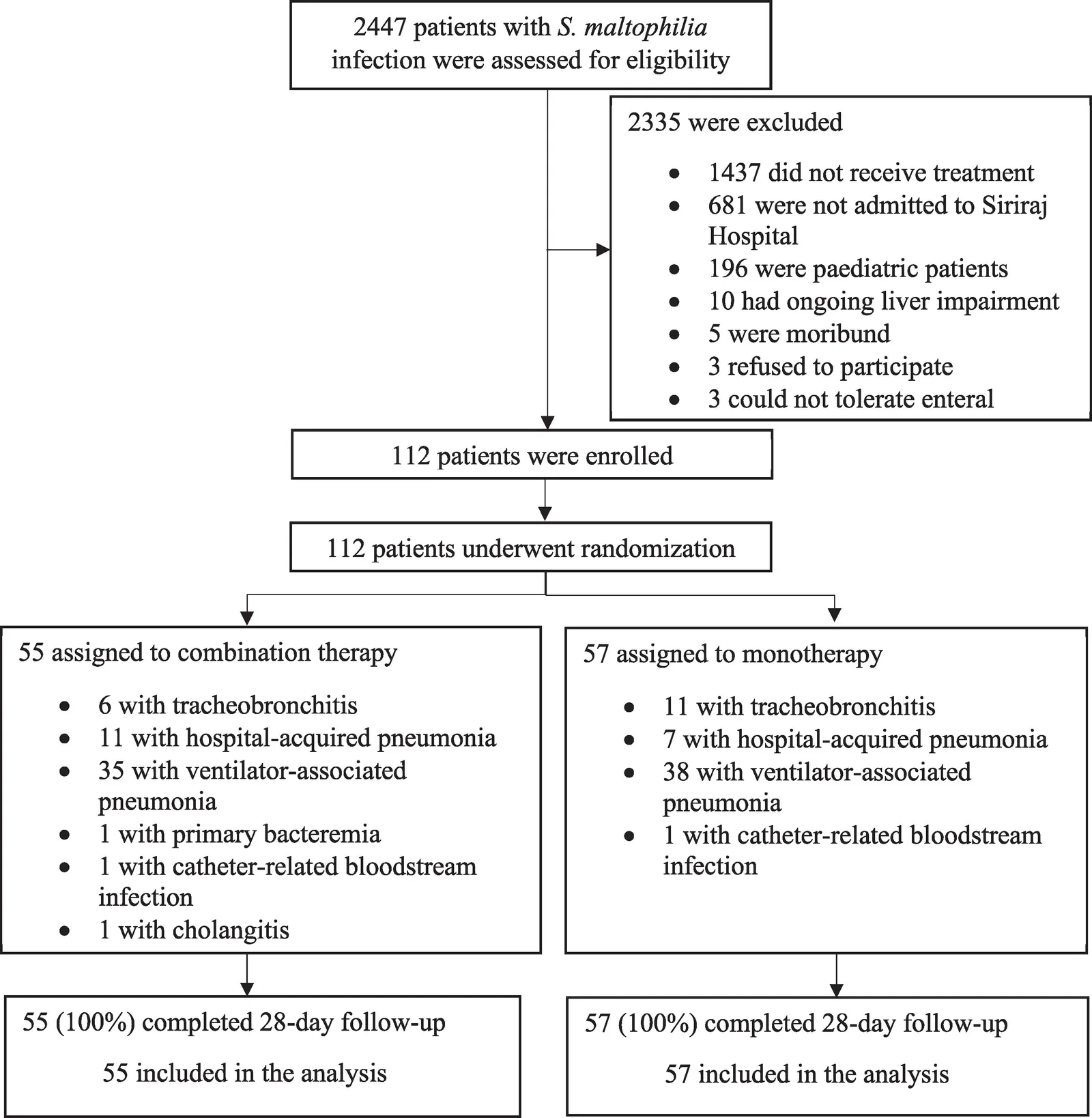
Consolidated standards of reporting trials flow diagram for enrolment in this trial.

Participants’ baseline characteristics were similar across groups (Table 1). Most participants were at an advanced age and had underlying medical conditions. In addition, most participants were on mechanical ventilation on the day of enrolment (90/112, 80.4%). The most common infection type was ventilator-associated pneumonia (VAP), which occurred at similar rates in both groups. Most participants received levofloxacin (100/112, 89.3%) as the first-line treatment for *S. maltophilia* infection. The median treatment duration was 10 (7–14) days, with similar treatment durations observed in both the combination therapy and monotherapy groups (10 [7–14] vs 9 [7–13] days, *P* = 0.420). A list of concurrent antibiotics used is provided in Table S3.

**Table 1.** Participants’ baseline characteristics in the entire cohort and in each group at enrolment

Characteristic	Total	Combination therapy	Monotherapy
	*n* = 112	*n* = 55	*n* = 57
Male sex, *n* (%)	71 (63.4%)	33 (60.0%)	38 (66.7%)
Age (years), mean ± SD	69 ± 15	68 ± 16	71 ± 13
Body mass index, mean ± SD	22.0 ± 5.0	23.0 ± 6.0	22.0 ± 4.0
Comorbidities, *n* (%)	110 (98.2%)	54 (98.2%)	56 (98.2%)
Diabetes mellitus	42 (37.5%)	23 (41.8%)	19 (33.3%)
COPD	8 (7.1%)	2 (3.6%)	6 (10.5%)
Chronic kidney disease	34 (30.4%)	18 (32.7%)	16 (33.3%)
Solid tumour	20 (17.9%)	6 (10.9%)	14 (24.6%)
Lymphoma	10 (8.9%)	5 (9.1%)	5 (8.8%)
Karnofsky performance status, median (IQR)	50 (40–80)	50 (40–90)	50 (40–70)
Charlson comorbidity index, median (IQR)	5 (3–7)	5 (3–7)	5 (3–7)
McCabe severity classification, *n* (%)			
Non-fatal	36 (32.1%)	16 (29.1%)	20 (35.1%)
Ultimately fatal	41 (36.6%)	22 (40.0%)	19 (33.3%)
Rapidly fatal	35 (31.3%)	17 (30.9%)	18 (31.6%)
qSOFA score, median (IQR)	2 (1–3)	3 (1–3)	2 (1–3)
ICU admission, *n* (%)	80 (71.4%)	39 (70.9%)	41 (71.9%)
History of antibiotic use before admission, *n* (%)	56 (50.0%)	27 (49.0%)	29 (50.9%)
Concurrent immunosuppressive use, *n* (%)	26 (23.2%)	17 (30.9%)	11 (19.3%)
Corticosteroids	18 (16.1%)	12 (21.8%)	6 (10.5%)
Other*	8 (7.1%)	5 (9.1%)	5 (9.1%)
Septic shock, *n* (%)	59 (52.7%)	30 (54.5%)	29 (50.9%)
Mechanical ventilation, *n* (%)	90 (80.4%)	45 (81.8%)	45 (78.9%)
Type of infection, *n* (%)			
VAP	74 (66.1%)	35 (63.6%)	39 (68.4%)
Hospital-acquired pneumonia	17 (15.2%)	11 (20.0%)	6 (10.5%)
Tracheobronchitis	17 (15.2%)	6 (10.9%)	11 (19.3%)
Primary bacteraemia	2 (1.8%)	2 (3.6%)	0
Catheter-related bloodstream infection	1 (0.9%)	0	1 (1.8%)
Cholangitis	1 (0.9%)	1 (1.8%)	0
Polymicrobial infection at presentation, *n* (%)	57 (50.9%)	29 (52.7%)	28 (49.1%)
First-line antibiotics received, *n* (%)			
Levofloxacin	100 (89.3%)	48 (87.3%)	52 (91.2%)
TMP/SMX	12 (10.7%)	7 (12.7%)	5 (8.8%)
Other concurrent antibiotic use, *n* (%)	109 (97.3%)	53 (96.4%)	56 (98.2%)
Treatment duration (days), median (IQR)	10 (7–14)	10 (7-−14)	9 (7–13)

A *P* value <0.05 was considered statistically significant.

COPD, chronic obstructive pulmonary disease; IQR, interquartile range; qSOFA, Quick Sequential Organ Failure Assessment; SLE, systemic lupus erythematosus; TMP/SMX, trimethoprim–sulfamethoxazole; VAP, ventilator-associated pneumonia.

### Participants’ clinical outcomes and microbial responses

Participants’ clinical outcomes and microbiological responses in the entire cohort and in each group are presented in Table 2. At 28 days, all-cause mortality occurred in 24 of 55 patients (43.6%) in the combination therapy group and 22 of 57 patients (38.6%) in the monotherapy group. The RR for 28-day mortality with combination therapy versus monotherapy was 1.13 (95% CI, 0.72–1.76), corresponding to an absolute RD of 5.0 percentage points (95% CI, −13.2 to 23.2; *P* = 0.590). Thus, adjunctive minocycline did not reduce 28-day mortality compared with monotherapy.

**Table 2.** Participants’ clinical outcomes and microbiological responses in the entire cohort and in each group

Parameter	Total	Combination therapy	Monotherapy	RD, percentage points/median difference^a^	*P* value	RR
	*n* = 112	*n* = 55	*n* = 57	(95% CI)		(95% CI)
First 72 h after study treatment initiation						
Favourable response	53 (47.3%)	29 (52.7%)	24 (42.1%)		0.260	
Unfavourable response	59 (52.7%)	26 (47.3%)	33 (57.9%)	−10.6 (−29.0, 7.8)		0.82 (0.57, 1.17)
End of study treatment						
Favourable response	69 (61.6%)	36 (65.5%)	33 (57.8%)		0.490	
Unfavourable response	33 (29.5%)	19 (34.5%)	24 (42.1%)	−7.6 (−25.5,10.4)		0.82 (0.51, 1.32)
First 72 h after study treatment initiation						
Eradication	33 (29.5%)	25 (45.5%)	8 (14.0%)		<0.001	
Persistence	74 (66.1%)	28 (50.9%)	46 (80.7%)	−29.8 (−46.5, −13.1)		0.63 (0.47, 0.84)
New pathogen	5 (4.5%)	2 (3.6%)	3 (5.3%)	−1.6 (−9.2, 6.0)		0.69 (0.12, 3.98)
End of study treatment						
Eradication	76 (67.9%)	44 (80.0%)	32 (56.1%)		0.023	
Persistence	27 (24.1%)	8 (14.5%)	19 (33.3%)	−18.8 (−34.2, −3.4)		0.44 (0.21, 0.91)
New pathogen	9 (8.0%)	3 (5.5%)	6 (10.5%)	−5.1 (−15.0, 4.9)		0.52 (0.14, 1.97)
28-day mortality, *n* (%)	46 (41.1%)	24 (43.6%)	22 (38.6%)	5 (−13.2, 23.2)^a^	0.590	1.13 (0.72, 1.76)
Hospital length of stay (days), median (IQR)	39 (25–62)	40 (25–69)	37 (25–56)	3 (−6, 14)^a^	0.920	
ICU length of stay (days), median (IQR)	20 (0–31)	16 (0–28)	22 (10–37)	−6 (−15, 0)^a^	0.098	

A *P* value <0.05 was considered statistically significant.

RD, absolute risk difference; RR, risk ratio; IQR, interquartile range.

^a^Hodges–Lehmann Median Difference.

The favourable clinical outcome rates were 47.3% and 61.6% at 72 h after the start of the study treatment and at the end of study treatment, respectively, in the entire cohort, and did not differ significantly between the combination therapy and monotherapy groups at either 72 h after the start of study treatment (52.7% vs 42.1%, *P* = 0.300) or the end of study treatment (65.5% vs 57.8%, *P* = 0.490). However, the microbiological response rate was significantly more favourable in the combination therapy group than in the monotherapy at both 72 h after the start of study treatment (45.5% vs 14.0%, *P* < 0.001) and at the end of study treatment (80.0% vs 56.1%, *P* = 0.023). At the end of study treatment, the rate of new pathogen acquisition was non-significantly higher in the monotherapy group than in the combination therapy group (10.5% vs 5.5%, *P* = 0.880). Finally, the median hospital length of stay was 39 (25–62) days in the entire cohort and did not differ significantly between groups (*P* = 0.920).

### Factors independently associated with 28-day all-cause mortality

The results of the univariate analysis to identify factors significantly and independently associated with 28-day all-cause mortality are presented in Table S4. After adjusting for ICU admission, history of antibiotic use before admission, other concurrent antibiotic use, concurrent immunosuppressive use, lymphoma, AKI, septic shock, ALT ≥30 IU, McCabe score and need for mechanical ventilation, the multivariate logistic regression analysis identified two factors independently associated with unfavourable clinical outcomes: history of antibiotic use before admission (aOR: 7.65, 95% CI: 2.04–28.76; *P* = 0.030) and septic shock (aOR: 7.24, 95% CI: 2.75–19.23; *P* < 0.001).

### Infections and antimicrobial resistance

The microbiological data are presented in Table S5. Polymicrobial infection at initial presentation was common in the trial cohort (*n* = 75, 67.0%). *Acinetobacter baumannii* was the most common coinfection (*n* = 36, 32.1%), followed by *Pseudomonas aeruginosa* (*n* = 21, 18.8%). Two participants in the combination therapy group developed intermediate resistance to levofloxacin, both of whom were treated with TMP/SMX. One participant in the monotherapy group had developed levofloxacin resistance at the end of study treatment. No participants developed resistance to TMP/SMX.

### Adverse events

The observed adverse events are listed in Table S6. AKI occurred in 65 (58.0%) participants, of whom 8 (12.5%) required subsequent haemodialysis. The AKI rates were similar in both groups. In all such cases, AKI was caused by septic shock and the concomitant administration of intravenous colistin, and the study drug was not considered causative. Hepatitis occurred in 10 participants in both groups. All such cases involved ischaemic hepatitis from ongoing septic shock, for which the study drug was not considered causative.

## Discussion

The current recommended standard of treatment for moderate-to-severe *S. maltophilia* infections varies across guidelines. For example, the Spanish Society of Infectious Diseases and Clinical Microbiology guidance recommends TMP/SMX monotherapy,^19^ while the 2024 IDSA guidance instead recommends combination therapy using two among TMP/SMX, minocycline/tigecycline, cefiderocol and levofloxacin, or the combination of ceftazidime–avibactam and aztreonam.^8^ Oral minocycline was used in this randomized controlled trial for various reasons. Firstly, it exhibits excellent bioavailability. Secondly, *in vitro* data suggest that it is the most effective drug against *S. maltophilia,*^20^ and it may have synergistic effects with other antibiotics.^21,22^ Finally, it demonstrates only minimal side effects.^23^ The 2024 IDSA guidance recommends a high minocycline dose of 200 mg twice daily. Notably, our trial was conducted before the 2024 IDSA guidance was published, and a dose half the amount recommended in that guidance but consistent with that in previous pharmacokinetic studies^12,24^ was expected to be sufficient to fully demonstrate the efficacy of combination therapy.

In our trial, most *S. maltophilia* infections occurred in the respiratory tracts of patients with significant comorbidities. Regarding *S. maltophilia* infection severity, septic shock occurred in about half of participants. In addition, 80% of participants were mechanically ventilated, and almost all received concurrent antibiotics. These findings are consistent with data from previously conducted cohort studies and case reports of patients with *S. maltophilia* infections, which reported that the major risk factors for acquiring such infections included mechanical ventilation and prior use of antibiotics such as carbapenems.^10,25,26^

In our trial, 28-day all-cause mortality did not differ significantly between the combination therapy and monotherapy groups (43.6% vs 38.6%, respectively; *P* = 0.590). The 28-day all-cause mortality rate in the overall cohort (41.1%) was comparable to rates reported in previous studies of hospitalized patients with *S. maltophilia* infections in the USA, South Korea and China.^26–29^ This finding is also consistent with a 2022 meta-analysis that found no definitive mortality benefit of combination therapy (overall effect estimate favouring combination therapy, 0.76; 95% CI, 0.18–3.18).^30^ However, these findings should be interpreted cautiously because the study was powered to detect a large treatment effect, corresponding to a 27-percentage-point absolute reduction in mortality, and may have been underpowered to detect smaller but potentially clinically meaningful differences in mortality. Therefore, the absence of a statistically significant between-group difference does not exclude more modest treatment effects.

Notably, the clinically favourable response rate was virtually identical in the combination therapy and monotherapy groups. In contrast, the favourable microbiological outcome rate was significantly higher in the combination therapy group than in the monotherapy group. The polymicrobial infection rate remained high throughout treatment. The most frequently co-isolated species was *A. baumannii* (32.1%). In contrast, a previous study conducted at Siriraj Hospital showed that Enterobacterales species were the most commonly isolated (38%).^10^ The post-treatment new pathogen acquisition rate was non-significantly higher in the monotherapy group than in the combination therapy group. Due to the complex nature of *S. maltophilia* infections in a tertiary care setting, the relative contributions of concurrent infections and antimicrobials used are difficult to determine, and they may have impacted the observed 28-day all-cause mortality rate.

Although patients who could not take or tolerate enteral nutrition (oral or tube feeding) were excluded from our trial, drug dosing at a level insufficient for treatment remains a concern and may have affected the obtained results. Immortal time bias is also a concern but should be negligible given the trial enrolment protocol. Notably, none of the observed adverse events was attributable to the study drug. The incidence of adverse events in our trial was relatively low compared with those in other reports.^31–33^

In the univariate analyses, the risk factors positively associated with 28-day all-cause mortality included ICU admission, a history of antibiotic use before admission, lymphoma and AKI. These risk factors for mortality were also reported in a 2018 study of patients with *S. maltophilia* bacteraemia.^34^ However, concurrent immunosuppressive use was not significantly associated with mortality in our trial, which contrasts with the findings of previous studies.^10,35^ In our multivariate logistic regression analysis, history of antibiotic use before admission and septic shock were significantly associated with 28-day all-cause mortality, similar to the results of previous studies.^36,37^

No cases of diarrhoea or *Clostridioides difficile* infection were observed in our trial cohort. Our antimicrobial susceptibility findings for all 112 *S. maltophilia* isolates were also consistent with previous studies,^2,25,28,38,39^ which reported slight to no resistance to minocycline. Thus, minocycline remains the most widely used drug to which *S. maltophilia* exhibits the highest susceptibility. Empirical treatment options for *S. maltophilia* infections at our hospital may include either minocycline or levofloxacin as alternatives. The observed levofloxacin resistance rate was lower among the 112 *S. maltophilia* isolates in our trial than globally.^40^ Emerging resistance to levofloxacin was observed in one participant. Further studies on the resistant *S. maltophilia* strain, including minimum inhibitory concentration tests and whole-genome sequencing, are planned to determine its possible mechanisms of resistance.

Our trial had several strengths, including its randomized, double-blind, placebo-controlled design and pragmatic approach. Previous studies have raised concerns regarding the accuracy of diagnosing true *S. maltophilia* infections.^10,18,41^ The prospective design enabled contemporaneous clinical assessment by the primary investigator and attending physician; however, it could not completely eliminate diagnostic uncertainty in distinguishing *S. maltophilia* pneumonia from respiratory tract colonization.

Several limitations should be acknowledged. First, distinguishing true *S. maltophilia* pneumonia from respiratory tract colonization is inherently challenging. Although the primary investigator and attending physician determined whether an isolate represented an infection requiring antimicrobial treatment based on the overall clinical context rather than culture results alone, no centrally adjudicated or fully standardized diagnostic criteria were used. Consequently, some patients with colonization may have been included. Such misclassification could have diluted between-group differences in clinical outcomes and biased the estimated treatment effect towards the null. Second, this was a single-centre study conducted at a tertiary care centre, which may limit the generalisability of the findings to other settings. Third, the study may have been underpowered because the observed reduction in mortality was lower than anticipated. Fourth, the minocycline dose used was lower than the IDSA-recommended dose. Finally, most infections involved the respiratory tract, which may limit the generalizability of our findings to other infection sites.

Nevertheless, our findings indicate that combination therapy with add-on oral minocycline did not confer an additional mortality benefit compared with the background monotherapy alone but did achieve higher favourable microbiological outcomes. Therefore, further randomized controlled trials examining a higher minocycline dose or conducting a head-to-head comparison of available treatment options are still needed to definitively identify the optimal treatment.

## Supplementary Material

dlag224_Supplementary_Data

## Supplementary data

Figure S1 and Tables S3–S6 are available as Supplementary data at *JAC-AMR* Online.
