# Supplementary material for: Efficacy of combination therapy with minocycline for treating Stenotrophomonas maltophilia infections
Source: JAC Antimicrob Resist. 2026 Oct 16;8(5):dlag224. doi: 10.1093/jacamr/dlag224 (PMC13673267; doi:10.1093/jacamr/dlag224)
Supplement: Supplementary file 1 [file dlag224_supplementary_data.zip › Supplementary_Appendix_AB_26_09_2026.docx]

**SUPPLEMENTARY APPENDIX**

**TABLE OF CONTENTS**

**1. SUPPLEMENTARY METHODS**

Blinding and concealment

Storage and accountability

Study population

Intervention

**2. SUPPLEMENTARY FIGURE
Figure S1:** A flow diagram of the trial protocol

**3. SUPPLEMENTARY TABLES
Table S1:** Trimethoprim dosage

**Table S2:** Levofloxacin dosage

**Table S3:** Concomitant antibiotics used

**Table S4:** Univariate analysis for factors independently associated with 28-day mortality

**Table S5:** Microbiological data of all patients

**Table S6:** Adverse events

**Table S7:** CONSORT 2010 checklist

**1. SUPPLEMENTARY METHODS**

**Blinding and concealment**

The investigator and attending physician were not aware of the group allocation after randomization. After randomization, each group was given an orange capsule as an add-on therapy (minocycline for the combination group and placebo for the monotherapy group). The capsule was identical for both groups and labelled either ‘group A’ or ‘group B’. The primary investigator performed the analysis, and the allocation group was not disclosed until the last patient to be enrolled had completed 28 days of follow-up.

**Storage and accountability**

The study drug was stored at room temperature in a sealed plastic bag labelled A or B. The package was stored in a locked, secured area with limited access. The investigator was responsible for maintaining a current record of inventory and drug accountability.

**Study population**

1. Intention-to-treat population – randomized group
2. Per-protocol population definition:
   2.1. Intervention – patients who received both the study drug and levofloxacin or trimethoprim/sulfamethoxazole for 7–28 days with simultaneous discontinuation of both drugs
   2.2. Control group – patients who received only levofloxacin or trimethoprim/sulfamethoxazole for 7–28 days

**Intervention**

**Add-on oral minocycline**

**Dosing:** a loading dose of 200 mg (four capsules) followed by a maintenance dose of 100 mg (two capsules) every 12 h.

Renal or hepatic adjustment is unnecessary

Minocycline was given at least 2 h apart from regular meals, antacids and polyvalent cation-containing supplements. The medication could be given via tube feeding.

Concomitantly prescribed antibiotics were not discontinued and were assessed solely by the primary attending physician. The investigators were not involved in any of the patient care processes.

3. **SUPPLEMENTARY FIGURE**

**Figure S1:** A flow diagram of the trial protocol

**3. SUPPLEMENTARY TABLES**

**Table S1:** Trimethoprim dosage

| **CrCl (mL/min)** | **Trimethoprim (TMP) dosage** |
| --- | --- |
| Normal renal function | 15–20 mg/kg/day divided every 6–12 h |
| > 50–90 | 5–20 mg/kg/day divided every 6–12 h |
| 30–50 | 5–20 mg/kg/day divided every 6–12 h |
| 10–29 | 5–10 mg/kg/day divided every 12 h |
| < 10 | 5–10 mg/kg/day every 24 h |
| Haemodialysis | 5–10 mg/kg/day every 24 h (dialysis day dose; given after dialysis) |
| CAPD | 5–10 mg/kg/day every 24 h |
| CRRT | 5 mg/kg/dose every 8 h |
| SLED | 5–10 mg/kg/day divided every 12 h |

Abbreviations: CAPD, continuous ambulatory peritoneal dialysis; CRRT, continuous renal replacement therapy; SLED, sustained low-efficacy dialysis.

**Table S2:** Levofloxacin dosage

| **CrCl (mL/min)** | **Levofloxacin dosage** |
| --- | --- |
| Normal renal function | 750 mg every 24 h |
| > 50–90 | 750 mg every 24 h |
| 20–49 | 750 mg every 48 h |
| < 20 | 750 mg for one dose, then 500 mg every 48 h |
| Haemodialysis | 750 mg for one dose, then 500 mg every 48 h |
| CAPD | 750 mg for one dose, then 500 mg every 48 h |
| CRRT | 750 mg for one dose, then 500 mg every 48 h |
| SLED | 750 mg every 48 h |

Abbreviations: CAPD, continuous ambulatory peritoneal dialysis; CRRT, continuous renal replacement therapy; SLED, sustained low-efficacy dialysis.

**Table S3:** Concomitant antibiotics used

| **Antibiotics** | **Number of patients** | | | |
| --- | --- | --- | --- | --- |
|  | **Total**  **(N = 112)** | **Combination therapy (N = 55)** | **Monotherapy (N = 57)** |  |
| Ceftriaxone | 6/112 (5.3%) | 4/55 (7.3%) | 2/57 (3.5%) |  |
| Ceftazidime | 10/112 (8.9%) | 6/55 (10.9%) | 4/57 (7.0%) |  |
| Cefoperazone/sulbactam | 3/112 (2.7%) | 1/55 (1.8%) | 2/57 (3.5%) |  |
| Piperacillin/tazobactam | 18/112 (16.1%) | 8/55 (14.5%) | 10/57 (17.5%) |  |
| Meropenem | 19/112 (17.0%) | 9/55 (16.4%) | 10/57 (17.5%) |  |
| Colistin | 38/112 (33.9%) | 20/55 (36.3%) | 18/57 (31.6%) |  |
| Fosfomycin (intravenous form) | 14/112 (12.5%) | 8/55 (14.5%) | 6/57 (10.5%) |  |
| Cloxacillin | 2/112 (1.8%) | 1/55 (1.8%) | 1/57 (1.8%) |  |
| Ceftaroline | 1/112 (0.9%) | 1/55 (1.8%) | 0/57 (0%) |  |
| Vancomycin (intravenous form) | 13/112 (11.6%) | 5/55 (9.1%) | 8/57 (14.0%) |  |
| Vancomycin (oral form) | 8/112 (7.1%) | 4/55 (7.3%) | 4/57 (7%) |  |
| Voriconazole | 12/112 (10.7%) | 7/55 (12.7%) | 5/57 (8.8%) |  |
| Micafungin | 4/112 (3.6%) | 3/55 (5.5%) | 1/57 (1.8%) |  |
| Amphotericin B deoxycholate | 3/112 (2.7%) | 2/55 (3.6%) | 1/57 (1.8%) |  |
| Ganciclovir | 5/112 (4.5%) | 2/55 (3.6%) | 3/57 (5.3%) |  |

**Table S4:** Univariate analysis for factors independently associated with 28-day mortality.

| Factors | Univariate analysis | | |
| --- | --- | --- | --- |
|  | Crude OR  (95% CI) | *p*-value |  |
| ICU admission | 3.57  (1.54–8.26) | 0.003 |  |
| History of antibiotic use before admission | 2.97  (1.07–8.28) | 0.037 |  |
| Received other antibiotics | 3.25  (1.47–7.18) | 0.004 |  |
| Received immunosuppressive drug | 1.70  (1.10–2.61) | 0.016 |  |
| Lymphoma | 1.68  (1.12–2.52) | 0.019 |  |
| Acute kidney injury | 2.83  (1.47–5.47) | 0.002 |  |
| Septic shock | 3.14  (1.72–5.73) | <0.001 |  |
| ALT ≥ 30 IU | 2.05  (1.23–3.41) | 0.006 |  |
| McCabe score | 3.50  (1.60–7.68) | 0.002 |  |
| Required mechanical ventilator | 4.50  (1.52–13.30) | 0.007 |  |

A *p*-value < 0.05 indicates statistical significance. Abbreviations: OR, odds ratio; CI, confidence interval.

**Table S5:** Microbiological data of all patients.

| Pathogen | Total | Combination therapy | Monotherapy | *p*-value |
| --- | --- | --- | --- | --- |
|  | N = 112 | N = 55 | N = 57 |  |
| Polymicrobial at initial presentation, *n* (%) | 57 (50.9%) | 29 (52.7%) | 28 (49.1%) | 0.71 |
| Polymicrobial at 72 h, *n* (%) | 48 (42.9%) | 21 (38.2%) | 27 (47.4%) | 0.33 |
| Polymicrobial at end of treatment, *n* (%) | 24 (21.4%) | 9 (16.4%) | 15 (26.3%) | 0.20 |
| Coinfection, *n* (%)  *Acinetobacter baumannii*  *Pseudomonas aeruginosa*  *Klebsiella pneumoniae*  MSSA  *Serratia marcescens*  Non-fermentative GNR | 36 (32.1%)  21 (18.8%)  3 (2.7%)  2 (1.8%)  1 (0.9%)  1 (0.9%) | 20 (36.4%)  8 (14.5%)  2 (3.6%)  0  1 (1.8%)  0 | 16 (28.1%)  13 (22.8%)  1 (1.8%)  2 (3.5%)  0  1 (1.8%) | 0.86 |
| Initial antimicrobial susceptibility, *n* (%) | | | | |
| Minocycline  Sensitive  Intermediate  Resistant | 100 (89.3%)  11 (9.8%)  1 (0.9%) | 48 (87.3%)  6 (10.9%)  1 (1.8%) | 52 (91.2%)  5 (8.8%)  0 | 0.47 |
| Levofloxacin  Sensitive  Intermediate  Resistant | 103 (92.0%)  2 (1.8%)  7 (6.3%) | 47 (85.5%)  2 (3.6%)  6 (10.9%) | 56 (98.2%)  0  1 (1.8%) |  |
| Trimethoprim–sulfamethoxazole  Sensitive  Intermediate  Resistant | 106 (94.6%)  0  6 (5.4%) | 51 (92.7%)  0  4 (7.3%) | 55 (96.5%)  0  2 (3.5%) |  |
| Emerging resistance, *n* (%) | | | | |
| Levofloxacin  Trimethoprim–sulfamethoxazole | 1 (0.9%)  0 | 0  0 | 1 (1.8%)  0 | 1.00 |

A *p*-value < 0.05 indicates statistical significance; Abbreviations: MSSA, methicillin-susceptible *Staphylococcus aureus*; GNR, gram-negative rods.

**Table S6:** Adverse events

| Adverse events | Total | Combination therapy | Monotherapy | *p*-value |
| --- | --- | --- | --- | --- |
|  | N = 112 | N = 55 | N = 57 |  |
| Total cases, *n* (%) | 52 (46.4%) | 28 (50.9%) | 24 (42.1%) | 0.80 |
| AKI (RIFLE^a^), *n* (%)  - Initiation of haemodialysis | 65 (58.0%)  14 (12.5%) | 34 (61.8%)  6 (10.9%) | 31 (54.4%)  8 (14.0%) | 0.31  0.42 |
| Hepatitis, *n* (%) | 10 (8.9%) | 6 (10.9%) | 4 (7.0%) | 0.47 |

^a^ RIFLE, risk, injury, failure, loss of kidney function and end-stage kidney disease. Abbreviation: AKI, acute kidney injury.

**Table S7:** CONSORT 2010 checklist

| **Section/Topic** | **Item No.** | **Checklist item** | **Page No.** |
| --- | --- | --- | --- |
| **Title and abstract** | | | |
|  | 1a | Identification as a randomized trial in the title | NA |
|  | 1b | Structured summary of trial design, methods, results and conclusions | NA |
| **Introduction** | | | |
| Background and objectives | 2a | Scientific background and explanation of rationale | 3 |
|  | 2b | Specific objectives or hypotheses | 4 |
| **Methods** | | | |
| Trial design | 3a | Description of trial design (such as parallel, factorial) including allocation ratio | 4 |
|  | 3b | Important changes to methods after trial commencement (such as eligibility criteria), with reasons | NA |
| Participants | 4a | Eligibility criteria for participants | 4-5 |
|  | 4b | Settings and locations where the data were collected | 4 |
| Interventions | 5 | The interventions for each group with sufficient details to allow replication, including how and when they were actually administered | 5 |
| Outcomes | 6a | Completely defined pre-specified primary and secondary outcome measures, including how and when they were assessed | 6-7 |
|  | 6b | Any changes to trial outcomes after the trial commenced, with reasons | NA |
| Sample size | 7a | How sample size was determined | 8 |
|  | 7b | When applicable, explanation of any interim analyses and stopping guidelines | NA |
| **Randomization** | | | |
| Sequence generation | 8a | Method used to generate the random allocation sequence | 5 |
|  | 8b | Type of randomization; details of any restriction (such as blocking and block size) | 5 |
| Allocation concealment mechanism | 9 | Mechanism used to implement the random allocation sequence (such as sequentially numbered containers), describing any steps taken to conceal the sequence until interventions were assigned | 5 |
| Implementation | 10 | Who generated the random allocation sequence, who enrolled participants, and who assigned participants to interventions | 5 |
| Blinding | 11a | If done, who was blinded after assignment to interventions (e.g. participants, care providers, those assessing outcomes) and how | 5 |
|  | 11b | If relevant, description of the similarity of interventions | 5-6 |
| Statistical methods | 12a | Statistical methods used to compare groups for primary and secondary outcomes | 7-8 |
|  | 12b | Methods for additional analyses, such as subgroup analyses and adjusted analyses | NA |
| **Results** | | | |
| Participant flow | 13a | For each group, the numbers of participants who were randomly assigned, received intended treatment and were analysed for the primary outcome | 9 |
|  | 13b | For each group, losses and exclusions after randomization, together with reasons | 9 |
| Recruitment | 14a | Dates defining the periods of recruitment and follow-up | 4 |
|  | 14b | Why the trial ended or was stopped | NA |
| Baseline data | 15 | A table showing baseline demographic and clinical characteristics for each group | 9 |
| Numbers analysed | 16 | For each group, number of participants (denominator) included in each analysis and whether the analysis was by original assigned groups | 9 |
| Outcomes  and estimation | 17a | For each primary and secondary outcome, results for each group, and the estimated effect size and its precision (such as 95% confidence interval) | 9-10 |
|  | 17b | For binary outcomes, presentation of both absolute and relative effect sizes is recommended | 9-10 |
| Ancillary analyses | 18 | Results of any other analyses performed, including subgroup analyses and adjusted analyses, distinguishing pre-specified from exploratory | NA |
| Harms | 19 | All important harms or unintended effects in each group | 11 |
| **Discussion** | | | |
| Limitations | 20 | Trial limitations, addressing sources of potential bias, imprecision, and, if relevant, multiplicity of analyses | 11-13 |
| Generalizability | 21 | Generalizability (external validity, applicability) of the trial findings | 14 |
| Interpretation | 22 | Interpretation consistent with results, balancing benefits and harms, and considering other relevant evidence | 14 |
| **Other information** | | | |
| Registration | 23 | Registration number and name of trial registry | 4 |
| Protocol | 24 | Where the full trial protocol can be accessed, if available | NA |
| Funding | 25 | Sources of funding and other support (such as supply of drugs), role of funders | 15 |

Abbreviations**:** No., number; NA, not applicable.
