# Supplementary material for: Efficacy of combination therapy with minocycline for treating Stenotrophomonas maltophilia infections
Source: JAC Antimicrob Resist. 2026 Oct 16;8(5):dlag224. doi: 10.1093/jacamr/dlag224 (PMC13673267; doi:10.1093/jacamr/dlag224)
Supplement: Supplementary file 1 [file dlag224_supplementary_data.zip › The full trial protocol_27_09_26.docx]

**Study Protocol**

This protocol has been provided by the authors to give readers additional information about their work.

Protocol for: Efficacy of combination therapy with minocycline for the treatment of *Stenotrophomonas maltophilia* infections. Open Forum Infect Dis. 2026 Jan 11;13(Suppl 1):ofaf695.1545. doi: 10.1093/ofid/ofaf695.1545. PMCID: PMC12792690.

**Protocol Summary**

**Title:** Efficacy of combination therapy with minocycline for treatment of *Stenotrophomonas maltophilia* infections

**Population:**

112 subjects who are greater than or equal to 18 to 95 years of age, are non-pregnant, and are in the inpatient setting of Siriraj Hospital, Thailand will be evaluated to treatment efficacy. Analysis will include subjects with any site of infection with *Stenotrophomonas maltophilia*.

**Number of Site:** 1

**Study Duration:** 2 years (November 2022 – November 2024)

**Subject Duration:** 28 days

**Objectives:**

Primary:

- To determine the efficacy of combination therapy with minocycline against *S. maltophilia* infections

Secondary:

- To determine clinical response and microbiological clearance following combination therapy
- To determine the microbiological susceptibility pattern of *S. maltophilia* strains in Siriraj Hospital, Thailand
- To determine the safety and adverse effects of minocycline, including
  - Nephrotoxicity
  - Hepatotoxicity

**1 BACKGROUND INFORMATION**

*Stenotrophomonas maltophilia* is a gram-negative bacterium that is a significant cause of hospital-acquired infections, especially in patients with multiple comorbidities (1). In hospitals, common sites of *S. maltophilia* infection include the bloodstream and lungs (2). Infections with *S. maltophilia* are associated with a high mortality rate, ranging from 21% to 69% (3).

A study conducted at Siriraj Hospital between 2007 and 2008 found that *S. maltophilia* was the third most common bloodstream infection (4). Risk factors for infection include broad-spectrum antibiotic use, prolonged hospitalisation, intensive care unit (ICU) stay, mechanical ventilation, central venous catheterisation and immunocompromised status (5, 6).

*S. maltophilia* generally exhibits multidrug resistance through various mechanisms such as reduced drug permeability, beta-lactamase production, carbapenemase and aminoglycoside-modifying enzymes, and drug efflux pumps. These contribute to poor treatment outcomes and high mortality rates (7, 8).

Treatment guidelines from the Infectious Diseases Society of America (IDSA)(9)and the European Society of Clinical Microbiology and Infectious Diseases (10) recommend using combination antibiotic therapy for severe infections, with trimethoprim-sulfamethoxazole as the mainstay of treatment. The IDSA 2024 Guidance states that minocycline is a preferred component of combination therapy due to its favourable in vitro characteristics, tolerability and the availability of susceptibility breakpoints. A study at Siriraj Hospital between 2013 and 2016 found that the susceptibility of *S. maltophilia* to minocycline, levofloxacin and trimethoprim-sulfamethoxazole was 100%, 94% and 91%, respectively (11). However, the study showed a mortality rate of 54% with the use of trimethoprim-sulfamethoxazole as the primary treatment, followed by levofloxacin as the alternative. Combination therapy was not generally practised in Siriraj Hospital, and minocycline was not available at the time of the study.

Although trimethoprim-sulfamethoxazole is the first-line treatment, an increasing trend of *S. maltophilia* resistance to this drug has been observed, with resistance rates ranging from 4% to 21% (12, 13). The dosage of trimethoprim-sulfamethoxazole required to treat *S. maltophilia* comprises a trimethoprim dose of 8 to 12 mg/kg/day (14, 15); more adverse events have been encountered with higher dosages. This has prompted the more frequent use of levofloxacin for the treatment of *S. maltophilia* in recent clinical practice (16). A study showed that *S. maltophilia* treatment with either trimethoprim-sulfamethoxazole or levofloxacin achieved similar efficacy (clinical cure rates of 61% and 52%, respectively). A 2023 meta-analysis showed that the worldwide prevalence of levofloxacin-resistant *S. maltophilia* was 14.4% (95% confidence interval (CI) = 12.5%–16.2%). The highest rate of trimethoprim-sulfamethoxazole resistance was observed in Asia, compared to Europe and America (17). Emerging resistances to treatment drugs were also noted in that study, with rates of 20% for trimethoprim-sulfamethoxazole and 30% for levofloxacin (18).

Minocycline, a tetracycline-class antibiotic, has been used since 1971 and is effective against a wide range of bacteria, including *S. maltophilia*. Minocycline is a protein synthesis inhibitor that binds to 30S and 50S bacterial ribosomal subunits. The drug is used for the treatment of respiratory and urinary tract infections (19). It is available in Thailand as 50 mg capsules and has good oral absorption, with a bioavailability of 95%–100% (20). No food-drug interactions occur with minocycline. An oral dosage of 200 mg achieves a peak concentration of 3.1 mg/dL within 2 hours (21), whereas an intravenous dosage of 200 mg achieves a peak concentration of 3.0 mg/dL. The average half-life values of the oral and intravenous forms of minocycline are comparable (11–22 hours and 15–23 hours, respectively) (22). Minocycline has high tissue penetration, especially in the lung tissue, with an expected lung tissue-to-serum concentration ratio of 3.8 (23).

Minocycline has minimal side effects. It may cause a photosensitive rash, dizziness, nausea, vomiting and diarrhoea. A study using minocycline as a treatment for severe infection reported no serious adverse effects (24). Minocycline is contraindicated in pregnant individuals and children under 8 years old due to tooth discolouration.

A systematic review of minocycline for lower respiratory tract infections found that it was effective in over 80% of cases (four studies with a total of 28 patients) (25). The dose of minocycline administered was 100 mg either orally or intravenously twice daily, and no serious adverse effect was reported.

According to the Clinical and Laboratory Standards Institute (CLSI) M100 34^th^ Edition guideline, the minimum inhibitory concentration susceptibility breakpoint of minocycline for *S. maltophilia* is less than or equal to 1 mcg/mL for the susceptible strain. Studies have shown a high susceptibility of *S. maltophilia* to minocycline, with a rate of 99.5%, compared to 93% for trimethoprim-sulfamethoxazole (26).

Despite its efficacy, only observational studies have addressed the use of minocycline combined with other antibiotics for the treatment of *S. maltophilia*. The mortality rate due to *S. maltophilia* infections at Siriraj Hospital of 54% (11) is higher than the rates reported in other countries, which range from 24% to 36.6% (5, 18).

**2 OBJECTIVES AND OUTCOME MEASURES**

**2.1 Objective 1 (Primary Objective)**

- Determine whether the treatment regimen of combination therapy with add-on minocycline is associated with a decreased risk of all-cause mortality during the 28-day post-enrollment period compared to either trimethoprim-sulfamethoxazole or levofloxacin alone for subjects with *S. maltophilia* infections in any site.

***Hypothesis 1***: Subjects with *S. maltophilia* infections treated with add-on minocycline will have a decreased risk for mortality compared to subjects treated with monotherapy.

**2.1.1 Primary Outcome Measure:** All-cause mortality

**2.2 Objective 2 (Secondary Objective)**

- Determine whether the treatment regimen of combination therapy with add-on minocycline is associated with improved clinical outcome or increase in microbiological clearance during the 28-day post-enrollment period compared to either trimethoprim-sulfamethoxazole or levofloxacin alone for subjects with *S. maltophilia* infections in any site.

***Hypothesis 2***: Subjects with *S. maltophilia* infections treated with add-on minocycline will have increased rate of favourable clinical outcome and increased microbiological clearance compared to subjects treated with monotherapy.

- Determine incidence of toxicities associated with minocycline

***Hypothesis 3:*** Minocycline therapy will have acceptable incidence of toxicities for clinical use.

**2.2.1 Secondary Outcome Measures**:

**2.2.1.1 Clinical outcome:**

Clinical assessment was performed daily until discontinuation of the study treatment or death or discharge from the hospital.

- Symptoms – both related and unrelated to the initial presentation
- Physical examination – signs present on initial presentation, vital signs and other relevant exams
- Adverse effects of treatment – if present, including diarrhoea, symptoms of hepatitis and acute kidney injury
- Signs and symptoms of presenting indications, which were monitored as shown

| **Presenting indication** | **Symptoms** | **Signs** |
| --- | --- | --- |
| Hospital-acquired pneumonia | - Cough - Dyspnoea | - Fever (oral or tympanic temperature ≥ 38 °C) - Hypothermia (oral or tympanic temperature < 35 °C) - Respiratory auscultations for abnormal signs such as rales and bronchial breath sounds |
| Ventilator-associated pneumonia  (mechanically ventilated) | - Purulent secretion | - Fever (oral or tympanic temperature ≥ 38 °C) - Hypothermia (oral or tympanic temperature < 35 °C) - Jaundice - Abdominal palpation - Bowel sound auscultation |
| Cholangitis | - Gastrointestinal symptoms such as nausea and vomiting - Abdominal tenderness - Feeding intolerance (if present) | - Fever (oral or tympanic temperature ≥ 38 °C) - Hypothermia (oral or tympanic temperature < 35 °C) - Jaundice - Abdominal palpation - Bowel sound auscultation |
| Bacteraemia |  | - Fever (oral or tympanic temperature ≥ 38 °C) - Hypothermia (oral or tympanic temperature < 35 °C) - Heart rate - Respiratory rate - Blood pressure |

Assessments were done at Day 3 after the initiation of the study treatment, at the end of the study treatment and 28 days after treatment with the study drug(s).

The clinical outcome was classified as follows:

- **Favourable outcome** – complete resolution or significant improvement of signs and symptoms that were present at the baseline and no new symptoms, such that no additional antimicrobial therapy is warranted
- **Unfavourable outcome** – symptoms present at study entry have not significantly improved or completely resolved, or new symptoms have developed and require the initiation of a non-study antimicrobial therapy; death or intolerance to the study drug leading to discontinuation of the study treatment.

**2.2.2.2 Microbiological clearance:**

**Microbiological assessment** was performed on Day 3 and the last day of treatment as follows:

- Gram staining of a specimen from the site of the initial infection
- Bacterial culture of a specimen from the site of the initial infection

**Microbiological outcomes** were assessed on Day 3 after the initiation of the study treatment and at the end of the study treatment. The microbiological outcomes were classified as follows:

- **Eradication** – the previously identified pathogen is not detected, and no new pathogen is detected
- **Persistence** – the same pathogen strain from the previous culture result is detected, with no new pathogen detected
- **New pathogen** – a pathogen species different from the previous culture result is detected

**2.2.2.3 Incidence of toxicities related to treatment medications:**

A variety of toxicity outcomes will be studied, including renal and hepatic.

**Renal impairment** – according to RIFLE (Risk, Injury, Failure, Loss of kidney function, and End-stage kidney disease) criteria

| Class | Glomerular filtration rate criteria | Urine output criteria |
| --- | --- | --- |
| Risk | Serum creatinine × 1.5 times | < 0.5 mL/kg/hour × 6 hours |
| Injury | Serum creatinine × 2.0 times | < 0.5 mL/kg/hour × 12 hours |
| Failure | Serum creatinine × 3.0 times or ≥ 4 mg/dL with an acute rise > 0.5 mg/dL | < 0.3 mL/kg/hour × 24 hours or anuria × 12 hours |
| Loss | Persistent acute renal failure, defined as a complete loss of kidney function > 4 weeks | |
| End-stage kidney disease | End-stage kidney disease > 3 months | |

Acute kidney injury in this study used the criteria in the ‘Injury’ row.

**Hepatitis** – confirmed AST or ALT elevation to five times the upper normal limit without symptoms or three times with symptoms (such as nausea, vomiting, fatigue, right upper quadrant pain) without alternative aetiology

**3 STUDY DESIGN**

This is a phase IV single-center, randomized, double-blind, placebo-controlled clinical study comparing two treatment regimens for *S. maltophilia* infections of any site.

Subjects will be randomized to Combination therapy group (add-on minocycline) or Monotherapy group (trimethoprim-sulfamethoxazole or levofloxacin). It is estimated that 124 subjects will participate in this study. The subject duration is expected to be 28 days and the study duration is expected to be 2 years.

All subjects wil be non-pregnant adult patients (≥18 to 95 years of age) enrolled in the inpatient setting in Siriraj Hospital, Thailand. Patients will have a microbiologically confirmed *S. maltophilia* infections that demonstrates in vitro sensitivity to minocycline. Detailed inclusion and exclusion criteria were provided in Section 4.

After study enrolment and randomization, subjects will receive one of two treatment groups medications. Both groups’ medications will be renally dosed.

In the monotherapy group, participants will receive levofloxacin 750 mg once daily, administered either orally as one and a half 500-mg tablets taken 30 minutes after a meal once daily, or intravenously once daily. Alternatively, participants may receive trimethoprim-sulfamethoxazole (TMP-SMX) at a dose of 15–20 mg/kg/day based on the trimethoprim component, administered either orally as tablets containing 80 mg of trimethoprim and 400 mg of sulfamethoxazole per tablet, or intravenously 2–3 times daily. Treatment will be administered continuously for 7–28 days, depending on the site and severity of the *S. maltophilia* infection.

Participants randomized to the two-agent therapy group will receive either trimethoprim-sulfamethoxazole or levofloxacin at the doses specified in combination with oral minocycline. Minocycline will be administered initially as a loading dose of 200 mg, followed by 100 mg twice daily, taken 2 hours after meals, for 7–28 consecutive days, depending on the site and severity of the *S. maltophilia* infection.

Participants will undergo repeat microbiological testing for S. maltophilia 3 days after initiation of treatment and at the end of treatment. All participants enrolled in the study will be closely monitored for both treatment efficacy and adverse effects. Clinical assessment, including review of symptoms and physical examination, will be performed daily. Blood tests to assess renal and hepatic function will be performed approximately twice weekly, with approximately 5 mL of blood collected at each assessment.

Detailed information on subject assessments and follow up was provided in Section 7.

**Schematic of Study Design**

| Screen/baseline | Treatment period | Follow-up | |
| --- | --- | --- | --- |
| Patients with  *S. maltophilia* infection  Randomise 1:1  Oral minocycline  100 mg PO q 12 hr  + LFX or TMP/SMX*  Oral placebo + LFX or TMP/SMX*  End of treatment  Assess survival  RANDOMISE | | | |
| Within 48 hours of effective prior therapy to the first dose of the study drug | Study Days 1 to 28 | Study Days 7 to 28 | Study Day 28 |

Abbreviations: PO, per oral; LFX, levofloxacin; TMP/SMX, trimethoprim-sulfamethoxazole.

**4 STUDY POPULATION**

**4.1 Inclusion/Exclusion Criteria**

**4.1.1 Inclusion criteria**

- Patients aged 18–95 years
- Patients admitted to Siriraj Hospital
- Patients with *Stenotrophomonas maltophilia* infection involving any organ or site, including patients with septic shock
- Patients who require treatment with a single active antimicrobial agent, either trimethoprim-sulfamethoxazole or levofloxacin, and who have received the antimicrobial agent for no more than 96 hours prior to enrolment
- Patients who are able to receive oral medication or medication administered via an enteral feeding tube
- Patients who agree to participate in the study after receiving a full explanation of the study from the investigator and having all questions or concerns adequately addressed

**4.1.2 Exclusion criteria**

- Pregnancy and lactation
- Patients with contraindications to minocycline, trimethoprim-sulfamethoxazole, or levofloxacin, such as a history of hypersensitivity or allergy to any of these medications
- Patients with active hepatitis

**4.2.3 Definitions of clinical syndromes**

**Diagnosis Definitions**

| Tracheobronchitis with *S.maltophilia* in respiratory samples | | |
| --- | --- | --- |
| All of the following: | **AND** signs or symptoms evidenced by at least two of the following: | **AND** at least one of the following: |
| 1. Absence of new or evolving infiltrate on chest X-ray, MRI, CT scan or ultrasound obtained within 48 hours before randomisation | 1. New onset of cough or worsening of baseline cough 2. Dyspnoea, tachypnoea or respiratory rate > 25 breaths/minute 3. Hypoxaemia (oxygen saturation < 90% or pO_2_ < 60 mmHg on ambient air or worsening of oxygen saturation or FiO_2_) 4. New onset of need for mechanical ventilation 5. Auscultatory findings consistent with pneumonia or pulmonary consolidation | - Fever (oral or tympanic temperature ≥ 38 °C) - Hypothermia (oral or tympanic temperature < 35 °C) - Elevated total peripheral WBC count (> 12,000 cells/mm^3^) - Leukopenia (total WBCs < 4000 cells/mm^3^) - > 10% immature neutrophils (bands) regardless of total WBC count |

| HAP with *S.maltophilia* in respiratory samples | | |
| --- | --- | --- |
| All of the following: | **AND** signs or symptoms evidenced by at least two of the following: | **AND** at least one of the following: |
| 1. Onset of symptoms > 48 hours after admission 2. Admission from long-term acute care, rehabilitation centre, hospital or skilled nursing home 3. New or evolving infiltrate on chest X-ray, MRI, CT scan or ultrasound obtained within 48 hours before randomisation | 1. New onset of cough or worsening of baseline cough 2. Dyspnoea, tachypnoea or respiratory rate > 25 breaths/minute 3. Hypoxaemia (oxygen saturation < 90% or pO_2_ < 60 mmHg on ambient air or worsening of oxygen saturation or FiO_2_) 4. New onset of need for mechanical ventilation 5. Auscultatory findings consistent with pneumonia or pulmonary consolidation | 1. Fever (oral or tympanic temperature ≥ 38 °C) 2. Hypothermia (oral or tympanic temperature < 35 °C) 3. Elevated total peripheral WBC count (> 12,000 cells/mm^3^) 4. Leukopenia (total WBCs < 4000 cells/mm^3^) 5. > 10% immature neutrophils (bands) regardless of total WBC count |

| VAP with *S.maltophilia* in respiratory samples | | |
| --- | --- | --- |
| All of the following: | **AND** signs or symptoms evidenced by at least two of the following: | **AND** at least one of the following: |
| 1. Onset of symptoms > 48 hours after receiving ventilator support via an endotracheal or nasotracheal tube 2. Requirement for ventilator support 3. New or evolving infiltrate on chest X-ray, MRI, CT scan or ultrasound obtained within 48 hours before randomisation | 1. Auscultatory findings consistent with pneumonia or pulmonary consolidation 2. Increased suctioning 3. Purulent tracheal aspirate 4. Acute change in ventilator support system to enhance oxygenation, determined by worsening oxygen saturation or oxygen saturation or FiO_2_ ratio | 1. Fever (oral or tympanic temperature ≥ 38 °C) 2. Hypothermia (oral or tympanic temperature < 35 °C) 3. Elevated total peripheral WBC count (> 12,000 cells/mm^3^) 4. Leukopenia (total WBCs < 4000 cells/mm^3^) 5. > 10% immature neutrophils (bands) regardless of total WBC count |

**Cholangitis with *S.maltophilia* in biliary tract samples**

A. Systemic inflammation

A-1 Fever and/or shaking chills

A-2 Laboratory evidence of an inflammatory response

B. Cholestasis

B-1 Jaundice

B-2 Abnormal liver function tests

C. Imaging

C-1 Biliary dilatation

C-2 Evidence of the aetiology on imaging (e.g., stricture, stone, stent)

Suspected diagnosis: one item in A + one item in either B or C

Definite diagnosis: one item in A, one item in B and one item in C

| **Primary bacteraemia with *S.maltophilia* (without identifiable source)** | |
| --- | --- |
| All of the following: | **AND** at least one of the following: |
| 1. Isolation of *S.maltophilia* from at least one blood culture collected from a peripheral vein | 1. Fever (oral or tympanic temperature ≥ 38 °C) 2. Hypothermia (oral or tympanic temperature < 35 °C) 3. Elevated total peripheral WBC count (> 10,000 cells/mm^3^) 4. Leukopenia (total WBCs < 4000 cells/mm^3^) 5. Tachycardia > 100 beats/minute 6. Tachypnoea > 25 breaths/minute 7. Hypotension, systolic blood pressure < 90 mmHg |

**SIRS, sepsis, septic shock**

SIRS – two or more of the following:

- Temperature > 38 °C or < 36 °C
- Heart rate > 90/min
- Respiratory rate > 20/min or PaCO_2_ < 32 mmHg (4.3 kPa)
- White blood cell count > 12,000 cells/mm^3^ or < 4000 cells/mm^3^ or > 10% immature bands

Sepsis – life-threatening organ dysfunction caused by a dysregulated host response to infection

- Acute change in total SOFA score ≥ 2 points consequent to the infection (suspected or documented infection)

| Sequential Organ Failure Assessment score | | | | | |
| --- | --- | --- | --- | --- | --- |
| Variables | SOFA score | | | | |
|  | 0 | 1 | 2 | 3 | 4 |
| Respiratory | PaO_2_/FiO_2_ > 400  SpO_2_/FiO_2_ > 302 | PaO_2_/FiO_2_ < 400  SpO_2_/FiO_2_ < 302 | PaO_2_/FiO_2_ < 300  SpO_2_/FiO_2_ < 221 | PaO_2_/FiO_2_ < 200  SpO_2_/FiO_2_ < 142 | PaO_2_/FiO_2_ < 100  SpO_2_/FiO_2_ < 67 |
| Cardiovascular  (doses in mcg/kg/min) | MAP ≥ 70 mmHg | MAP ≥ 70 mmHg | Dopamine ≤ 5 or ANY dobutamine | Dopamine > 5  Norepinephrine ≤ 0.1  Phenylephrine ≤ 0.8 | Dopamine > 15  Norepinephrine > 0.1  Phenylephrine > 0.8 |
| Liver  (bilirubin, mg/dL) | < 1.2 | 1.2–1.9 | 2.0–5.9 | 6.0–11.9 | > 12 |
| Renal  (creatinine, mg/dL) | < 1.2 | 1.2–1.9 | 2.0–3.4 | 3.5–4.9 | > 5.0 |
| Coagulation  (platelets × 10^3^/mm^3^) | ≥ 150 | < 150 | < 100 | < 50 | < 20 |
| Neurologic  (GCS score) | 15 | 13–14 | 10–12 | 6–9 | < 6 |

- Septic shock – a clinical construct of sepsis with persistent hypotension, requiring vasopressor therapy to elevate mean arterial pressure to 65 mmHg and having serum lactate > 2 mmol/L (18 mg/dL) despite adequate fluid resuscitation

**5 STUDY MEDICATIONS, DOSING AND BLINDING PROCEDURE**

**5.1 Study Arms**

Combination therapy:	Minocycline and Trimethoprim-sulfamethoxazole or Levofloxacin
Monotherapy:	Placebo and Trimethoprim-sulfamethoxazole or Levofloxacin

**5.2 Trimethoprim-sulfamethoxazole (TMP-SMX)**

**5.2.1 Preparation**

TMP-SMX will be administered in the form of diluted reconstituted intravenous solution or tablet.

TMP-SMX tablet is available in regular strength which contains 80 mg of trimethoprim and 400 mg of sulfamethoxazole.

Intravenous form is available in ampoule. Each 5 mL ampoule contains 80 mg trimethoprim and 400 mg sulfamethoxazole. Each ampoule is diluted in 5% dextrose in sterile water of 125 mL. It is stored at room temperature and used within 6 hours after reconstitution.

In settings of fluid overload, determinations to dilute meropenem in a smaller volume of 5% dextrose in sterile water can be made on a case-by-case basis through discussions with principal investigator and primary physician.

**5.2.2 Administration and Dosage**

TMP-SMX will be provided in both of the treatment arms, at the standardized dose of TMP 15-20 mg/kg/day divided every 6-12 hours and infused over 60 to 90 minutes.

If the subject’s actual body weight is less than their ideal body weight (IBW), use the actual body weight (ABW).

If the subject’s actual body weight is more than 130% of their ideal body weight (IBW), use the adjusted body weight (AdjBW) using the following equation:

For men: IBW (kg) = 50 + 2.3 x height in inches – 60

For women: IBW (kg) = 45.5 + 2.3 x height in inches - 60

AdjBW = IBW + 0.4 x (ABW – IBW)

TMP-SMX will be adjusted for renal insufficiency according to the following table adapted from the local institution recommendations.

| **CrCl (mL/min)** | **Trimethoprim (TMP) dosage** |
| --- | --- |
| Normal renal function | 15–20 mg/kg/day divided every 6–12 hours |
| > 50–90 | 5–20 mg/kg/day divided every 6–12 hours |
| 30–50 | 5–20 mg/kg/day divided every 6–12 hours |
| 10–29 | 5–10 mg/kg/day divided every 12 hours |
| < 10 | 5–10 mg/kg/day every 24 hours |
| Haemodialysis | 5–10 mg/kg/day every 24 hours (dialysis day dose; given after dialysis) |
| CAPD | 5–10 mg/kg/day every 24 hours |
| CRRT | 5 mg/kg/dose every 8 hours |
| SLED | 5–10 mg/kg/day divided every 12 hours |

**5.3 Levofloxacin**

**5.3.1 Preparation**

Levofloxacin will be administered in the form of intravenous infusion formulations or tablets.

Levofloxacin tablet is available in 500 mg strength and intravenous form is available in premixed solution with a concentration of 750 mg/150 mL.

**5.3.2 Administration and Dosage**

Levofloxacin will be provided in both of the treatment arms, at the standardized dose of 750 mg per day. If intravenous form is given, it is infused over 90 minutes.

Oral dosing with tablets can be without regard to meals. Subjects are instructed to avoid using antacids, or levofloxacin should be administered 2 hours before or 2 hours after taking antacids containing magnesium or aluminum.

Levofloxacin will be adjusted for renal insufficiency according to the following table adapted from the local institution recommendations.

| **CrCl (mL/min)** | **Levofloxacin dosage** |
| --- | --- |
| Normal renal function | 750 mg every 24 hours |
| > 50–90 | 750 mg every 24 hours |
| 20–49 | 750 mg every 48 hours |
| < 20 | 750 mg for one dose, then 500 mg every 48 hours |
| Hemodialysis | 750 mg for one dose, then 500 mg every 48 hours |
| CAPD | 750 mg for one dose, then 500 mg every 48 hours |
| CRRT | 750 mg for one dose, then 500 mg every 48 hours |
| SLED | 750 mg every 48 hours |

**5.4 Minocycline**

**5.4.1 Preparation**

Minocycline is available in oral capsule form in Thailand in 50 mg per capsule strength formulation.

**5.4.2 Administration and Dosage**

Minocycline will be provided in only the combination therapy group as an add-on therapy to baseline TMP-SMX or Levofloxacin.

Minocycline will be given at the standardized loading dose of 4 capsules (200 mg) then followed by 2 capsules (100 mg) every 12 hours.

No dose adjustment of minocycline is required in subjects with impaired renal or hepatic function.

Food may delay the time required for minocycline to reach its peak serum concentration. Subjects are instructed to avoid taking minocycline concomitantly with food. If subjects are taking calcium, iron, aluminum-containing products, liquid antacids, or milk, minocycline is administered at least 2 hours apart from these products to avoid interaction.

**5.5 Placebo**

A placebo will be used to mimic the appearance of minocycline by utilizing orange capsule with the same size and shape to minocycline capsule. Dosing regimen is the same as minocycline with the loading dose of 4 capsules and maintenance dose of 2 capsules every 12 hours. No dose adjustment is required in subjects with impaired renal or hepatic function.

**5.6 Accountability of Study Medications**

Accountability and supply of all study medications (TMP-SMX and Levofloxacin) will be maintained by Siriraj Hospital, Thailand. Minocycline is provided from Qualimed Co., Ltd. (Thailand) in a sealed container with humidity adsorbent and documenting study medication supplies in the accountability logs.

**5.7 Final Disposition of Study Medications**

At the completion of the study and the final visit by principal investigator or co-investigator, or in the instance of expired medication, all unused medication should be destroyed in accordance with Siriraj institutional policy.

**5.8 Blinding, Preparation and Labeling of Study Medications**

Study medications will be prepared by the designated research pharmacist. Study medication will be prepared in accordance with the study protocol and package insert following standard procedures within Siriraj Hospital.

TMP-SMX and Levofloxacin will not be blinded.

Minocycline and placebo will be blinded active drugs in this study. The study drugs will be given in a plastic bag container with labeling as A or B in the front.

Principal investigator and co-investigator will not be aware of the treatment allocation of each participant until the end of the study period.

**6 STUDY PROCEDURES, RANDOMIZATION AND EVALUATIONS**

**6.1 Study Procedures and Randomization**

Site principal investigator (PI) and co-investigators (CI) will review culture result from patients that grow *S. maltophilia*. The physician of record will be contacted by study PI or CI regarding the potential participation of their patient in the study.

All clinical data (medical history, physical exams, concomitant medications, etc.) and laboratory data required for verification of eligibility of subjects must be available in the subjects’ chart.

**Randomization**:

Subjects who met all predefined eligibility criteria were randomly assigned in a 1:1 ratio to receive either placebo or minocycline using block randomization with a block size of four. The randomization sequence was generated using a web-based randomization system (Sealed Envelope Ltd., 2022). Following randomization, the allocation sequence was concealed in opaque, sealed envelopes. The subjects, investigators, and treating physicians were blinded to treatment allocation. After the investigator opened the sealed envelope and determined the treatment assignment, the subject received the treatment corresponding to the assigned study group.

**7 STUDY SCHEDULE**

**7.1 Screening/Baseline**

PI and CI who are Infectious Disease physicians will review daily culture results from the clinical microbiology laboratory at Siriraj Hospital. PI or CI will visit the subject to verify the eligibility to enrol into the study using pre-specified clinical and laboratory criteria for diagnosis of *S. maltophilia* infection. Contact with primary physician responsible for the subject will be made to inform of the enrolment into the study.

**7.2 Enrolment, Follow-up and Final Visits**

If the subject meets the eligibility criteria stated and are not excluded. The subjects will be enrolled onto the study and will be randomized.

Study schedule is provided in the table below:

| Procedure | Screening | Treatment Period | | | | | | EOT | Last follow-up |
| --- | --- | --- | --- | --- | --- | --- | --- | --- | --- |
|  | -96 hours to Day 1 | D1 | D2 | D3 | D4 | D5 | D6 | Day 7 to 28 | Day 28 |
| Informed consent | X |  |  |  |  |  |  |  |  |
| Inclusion/exclusion criteria | X |  |  |  |  |  |  |  |  |
| Medical/surgical history | X |  |  |  |  |  |  |  |  |
| Prior/concomitant medications | X | X | X | X | X | X | X | X |  |
| Demographics | X |  |  |  |  |  |  |  |  |
| Physical examination | X | X | X | X | X | X | X | X | X |
| Vital signs | X | X | X | X | X | X | X | X | X |
| Randomisation |  | X |  |  |  |  |  |  |  |
| Serum liver function test^a^ |  |  |  | X |  |  |  | X |  |
| Serum creatinine^a^ |  |  |  | X |  |  |  | X |  |
| Chest X-ray (if pneumonia) | X |  |  | X |  |  |  | X |  |
| Administer study drug |  | X | X | X | X | X | X | X |  |
| Assess clinical signs/symptoms |  | X | X | X | X | X | X |  | X |
| Assess clinical outcomes |  |  |  | X |  |  |  | X | X |
| Infection site-specific cultures^b^ | X |  |  | X |  |  |  | X |  |
| Assessment of adverse events |  | X | X | X | X | X | X | X |  |

^a^ done two times per week of study drug treatment; ^b^ same site(s) as initial presenting diagnosis.

Abbreviations: D, day; EOT, end of treatment; qSOFA, Quick Sequential Organ Failure Assessment.

**7.3 Criteria for Discontinuation or Withdrawal of a Subject**

Participants may be withdrawn from the study under the following circumstances:

- The participant withdraws their consent or no longer wishes to continue participating in the study
- In participants who develop ileus after receiving minocycline, withdrawal from the study will be considered if the gastric residual volume (GRV) is ≥500 mL after enteral feeding has been withheld for at least 2 hours
- The participant develops adverse effects potentially attributable to minocycline, including:
  - Hepatitis (incidence <1%)
  - Diarrhea (incidence approximately 1%)
  - Renal failure (incidence <1%)

These criteria do not include patients with pre-existing cirrhosis at any stage or chronic kidney disease at any stage, as minocycline does not require dose adjustment based on impaired hepatic or renal function

**8 SAFETY ASSESSMENT AND REPORTING**

**8.1 Definition of Adverse Event (AE)**

Any untoward or unfavourable medical occurrence in a human subject, including any abnormal sign, symptom, or disease, temporally associated with the subject’s participation in the research.

In this study only events that represent worsening of medical conditions that are related to the study outcomes, including nephrotoxicity, hepatotoxicity will be collected as AEs (Definitions in Section 2.2.2.3).

**8.2 Data Recording and Reporting Procedures**

The occurrence of an AE may come to the attention of study personnel during study visits or interactions with the subject, during the monitoring of the subjects’ labs, or upon review of the subject’s data by PI or CI.

AEs must be recorded on case study record form under section adverse events. Information to be collected includes event description, time of onset, clinician’s assessment of severity, relationship to study product, and date of resolution.

Subjects will be continuously monitored for AEs from time of enrolment until EOT.

**9 STATISTICAL CONSIDERATIONS**

**9.1 Sample Size Considerations**

In a retrospective study in Siriraj Hospital, the in-hospital mortality rate of patients with *S. maltophilia* infection treated with antibiotic monotherapy was 54% (11). In comparison, in a study by Shah et al., combination therapy achieved a 30-day infection-related mortality of 15.8% (34). Assuming that combination therapy will decrease the mortality rate by 50% (from 54% to 27%), and allowing for a 5% type I error and a 20% type II error, the sample size calculation generated a minimum of 51 patients per group. The formula used is shown below.

Formula used for sample size calculation

n denotes the population size in each group

p_1_ denotes the probability of mortality in the monotherapy group, which equals 54% (0.54).

p_2_ denotes the probability of mortality in the combination therapy group, which equals 27% (0.27).

Z_α_ denotes type I error, which equals 1.96 (at 5% type I error).

Z_ß_ denotes type II error, which equals 0.84 (at 20% type II error).

To account for possible dropouts, the sample size was increased by 20% in each group. A total of 62 patients were required in each group. The total sample size for this study was 124 patients.

**9.2 Analysis Plan**

The project statistician will be blinded to which treatment group is which and will have responsibility for the final analyses for efficacy, secondary outcomes, and for toxicity.

The data were compared using the following tests:

- Categorical data: chi-squared test or Fischer’s exact test
- Continuous data:
  - Normally distributed data – Student’s t-test
  - Non-normally distributed data – Mann–Whitney U test.

The results of these comparisons are reported as number and percentage for categorical data and mean ± standard deviation (SD), and median and interquartile range (IQR) for normally distributed and non-normally distributed continuous data, respectively.

Univariate and multivariate analyses were performed to identify the risk factors significantly and independently associated with 28-day mortality. A multivariate analysis was performed using logistic regression. The univariate analysis results are given as the crude odds ratio (OR) and 95% CI. The multivariate analysis results are given as the adjusted OR and 95% CI. Intention-to-treat and per-protocol analyses were done according to the definition given in each group and are reported separately. A p-value of < 0.05 was considered statistically significant for all tests. All statistical analyses were performed using the SPSS Statistics for Windows software package, version 20 (IBM Corp., Armonk, NY, USA).

**10 DATA HANDLING AND RECORD KEEPING**

**10.1 Data Management Responsibilities**

PI and CI will be responsible for data collection and management.

Case record forms (CRFs) with subjects’ ID will be developed. Primary responsibility for CRF content will rest with the study investigators.

**10.2 Study Records Retention**

Personal information will be stored on a password-protected personal computer to prevent unauthorized access. Documents, CDs, and electronic files containing personal information will be securely stored in a locked cabinet or drawer, with access restricted to the investigators who possess the key. All study-related documents and electronic data will be retained for 1 year after completion of the study.

No study documents will be destroyed without authorization from PI.

**11 ETHICAL CONSIDERATIONS**

The site Institutional Review Boards (IRBs) ensure the safe and ethical conduct of human participant research. Characteristics of research participants were:

- Patients in the Emergency Department or Intensive Care Unit (ICU), and patients receiving end-of-life care

- Patients with chronic illnesses who are dependent on physicians and caregivers

For participants considered vulnerable subjects*, informed consent will be obtained from their legally authorized representatives (LARs). The legally authorized representative will be approached in the following order of priority: spouse, children, parents, full siblings, half-siblings, grandparents, and aunts/uncles. The legally authorized representative will be asked to provide informed consent by signing the Informed Consent Form for participation in the research study.

**11.1 Informed Consent Process**

The principal investigator or co-investigators will provide information about the study and obtain informed consent from eligible subjects. The initial discussion will take approximately 5–10 minutes, followed by approximately 1 hour for the informed consent process. The study will be explained in simple, understandable Thai. Medical terminology will be explained in plain language to ensure that subjects or their legally authorized representatives fully understand the information provided.

For subjects who are critically ill or comatose and unable to provide informed consent, consent will be obtained from a legally authorized representative. The legally authorized representative will be approached in the following order: spouse, children, parents, full siblings, half-siblings, grandparents, and aunts or uncles. The representative will be provided with the Patient Information Sheet and given sufficient time to review the information, discuss the study with other family members or caregivers, and ask any questions they may have. The investigator will subsequently follow up with the subject or representative regarding the decision to participate. If the subject is unable to provide consent at the time of enrollment but subsequently regains sufficient capacity to make decisions, the subject will be informed about the study and asked to provide consent to continue participation.

Informed consent will be obtained in a private and appropriate setting at the inpatient ward of Siriraj Hospital. The investigator will explain the study according to the information provided in the Patient Information Sheet, which will be given to the subject or representative for further review. Subjects or their representatives will be informed that they may contact the investigator using the contact information provided in the Patient Information Sheet if they have any questions or concerns. If they agree to participate, the subject or legally authorized representative will sign the informed consent form before proceeding to the study screening process. Throughout the informed consent process, the privacy of the subject and confidentiality of personal information will be maintained.

**12 LITERATURE REFERENCES**

1.	Brooke JS. Stenotrophomonas maltophilia: an emerging global opportunistic pathogen. Clin Microbiol Rev. 2012;25(1):2-41.

2.	Looney WJ, Narita M, Mühlemann K. Stenotrophomonas maltophilia: an emerging opportunist human pathogen. The Lancet infectious diseases. 2009;9(5):312-23.

3.	Paez JG, Costa S. Risk factors associated with mortality of infections caused by Stenotrophomonas maltophilia: a systematic review. Journal of Hospital Infection. 2008;70(2):101-8.

4.	Rattanaumpawan P, Ussavasodhi P, Kiratisin P, Aswapokee N. Epidemiology of bacteremia caused by uncommon non-fermentative gram-negative bacteria. BMC infectious diseases. 2013;13:1-8.

5.	Jeon YD, Jeong WY, Kim MH, Jung IY, Ahn MY, Ann HW, et al. Risk factors for mortality in patients with Stenotrophomonas maltophilia bacteremia. Medicine. 2016;95(31):e4375.

6.	Xun M, Zhang Y, Li B-L, Wu M, Zong Y, Yin Y-M. Clinical characteristics and risk factors of infections caused by Stenotrophomonas maltophilia in a hospital in northwest China. The Journal of Infection in Developing Countries. 2014;8(08):1000-5.

7.	Vila J, Marco F. Interpretive reading of the non-fermenting gram-negative bacilli antibiogram. Enfermedades Infecciosas y Microbiología Clínica. 2010;28(10):726-36.

8.	Sanchez MB, Hernandez A, Martinez JL. Stenotrophomonas maltophilia drug resistance. Future microbiology. 2009;4(6):655-60.

9.	Tamma PD, Heil EL, Justo JA, Mathers AJ, Satlin MJ, Bonomo RA. Infectious Diseases Society of America 2024 Guidance on the Treatment of Antimicrobial-Resistant Gram-Negative Infections. Clin Infect Dis. 2024.

10.	Paul M, Carrara E, Retamar P, Tängdén T, Bitterman R, Bonomo RA, et al. European Society of Clinical Microbiology and Infectious Diseases (ESCMID) guidelines for the treatment of infections caused by multidrug-resistant Gram-negative bacilli (endorsed by European society of intensive care medicine). Clinical Microbiology and Infection. 2022;28(4):521-47.

11.	Insuwanno W, Kiratisin P, Jitmuang A. Stenotrophomonas maltophilia Infections: Clinical Characteristics and Factors Associated with Mortality of Hospitalized Patients. Infect Drug Resist. 2020;13:1559-66.

12.	Gales AC, Seifert H, Gur D, Castanheira M, Jones RN, Sader HS, editors. Antimicrobial susceptibility of Acinetobacter calcoaceticus–Acinetobacter baumannii complex and Stenotrophomonas maltophilia clinical isolates: results from the SENTRY antimicrobial surveillance program (1997–2016). Open forum infectious diseases; 2019: Oxford University Press US.

13.	Brooke JS. Advances in the microbiology of Stenotrophomonas maltophilia. Clinical microbiology reviews. 2021;34(3):10.1128/cmr. 00030-19.

14.	Junco SJ, Bowman MC, Turner RB. Clinical outcomes of Stenotrophomonas maltophilia infection treated with trimethoprim/sulfamethoxazole, minocycline, or fluoroquinolone monotherapy. International journal of antimicrobial agents. 2021;58(2):106367.

15.	Tamma PD, Aitken SL, Bonomo RA, Mathers AJ, van Duin D, Clancy CJ. Infectious Diseases Society of America 2023 Guidance on the Treatment of Antimicrobial Resistant Gram-Negative Infections. Clin Infect Dis. 2023.

16.	Nys C, Cherabuddi K, Venugopalan V, Klinker KP. Clinical and microbiologic outcomes in patients with monomicrobial Stenotrophomonas maltophilia infections. Antimicrobial Agents and Chemotherapy. 2019;63(11):10.1128/aac. 00788-19.

17.	Masoud D, Bahareh H, Nooshin N, Negin N, Shahrooz Y, Ali H, et al. Global prevalence and distribution of antibiotic resistance among clinical isolates of Stenotrophomonas maltophilia: A systematic review and meta-analysis. Journal of Global Antimicrobial Resistance. 2023;34:253-67.

18.	Wang YL, Scipione MR, Dubrovskaya Y, Papadopoulos J. Monotherapy with fluoroquinolone or trimethoprim-sulfamethoxazole for treatment of Stenotrophomonas maltophilia infections. Antimicrobial agents and chemotherapy. 2014;58(1):176-82.

19.	Agwuh KN, MacGowan A. Pharmacokinetics and pharmacodynamics of the tetracyclines including glycylcyclines. Journal of Antimicrobial Chemotherapy. 2006;58(2):256-65.

20.	Saivin S, Houin G. Clinical pharmacokinetics of doxycycline and minocycline. Clinical pharmacokinetics. 1988;15:355-66.

21.	Wood M, Farrell W, Kattan S, Williams J. Activity of minocycline and tetracycline against respiratory pathogens related to blood levels. Journal of Antimicrobial Chemotherapy. 1975;1(3):323-31.

22.	Simon C, Malerczyk V, Preuss I, Schmidt K, Grahmann H. Activity in vitro and pharmacokinetics of minocycline (author's transl). Arzneimittel-forschung. 1976;26(4):556-60.

23.	Colton B, McConeghy KW, Schreckenberger PC, Danziger LH. IV minocycline revisited for infections caused by multidrug-resistant organisms. American journal of health-system pharmacy. 2016;73(5):279-85.

24.	Rogers J, Barnwell P, Waterman N, Austin F, Raff M. Clinical evaluation of intravenous minocycline. International Journal of Clinical Pharmacology and Biopharmacy. 1977;15(4):194-8.

25.	Ritchie DJ, Garavaglia-Wilson A. A review of intravenous minocycline for treatment of multidrug-resistant Acinetobacter infections. Clinical infectious diseases. 2014;59(suppl_6):S374-S80.

26.	Flamm RK, Shortridge D, Castanheira M, Sader HS, Pfaller MA. In vitro activity of minocycline against US isolates of Acinetobacter baumannii-Acinetobacter calcoaceticus species complex, Stenotrophomonas maltophilia, and Burkholderia cepacia complex: results from the SENTRY Antimicrobial Surveillance Program, 2014 to 2018. Antimicrobial Agents and Chemotherapy. 2019;63(11):10.1128/aac. 01154-19.
